# Genetic Testing for Primary Aldosteronism in SPAIN: Results From the SPAIN-ALDO Registry and Review of the Literature

**DOI:** 10.1210/clinem/dgae523

**Published:** 2024-07-26

**Authors:** Marta Araujo-Castro, Jorge Gabriel Ruiz-Sánchez, César Gonzalvo, Cristina Lamas, Paola Parra Ramírez, Patricia Martín Marcos-Rojas, Miguel Paja, Cristina Robles Lázaro, Theodora Michalopoulou, María Tous, M Gonzalez-Boillos, José María Recio-Córdova, Anna Casteras, Paula Fernández-Álvarez, Verónica Barca Tierno, Paolo Mulatero

**Affiliations:** Endocrinology & Nutrition Department, Hospital Universitario Ramón y Cajal, 28034 Madrid, Spain; Instituto de Investigación Biomédica Ramón y Cajal (IRYCIS), 28034 Madrid, Spain; Endocrinology & Nutrition Department, Instituto de Investigación Sanitaria Fundación Jiménez Díaz (IIS FJD, UAM), Hospital Universitario Fundación Jiménez Díaz, 28040 Madrid, Spain; Endocrinology & Nutrition Department, Hospital Universitario de Albacete, 02006 Albacete, Spain; Endocrinology & Nutrition Department, Hospital Universitario de Albacete, 02006 Albacete, Spain; Endocrinology & Nutrition Department, Hospital Universitario La Paz, 28046 Madrid, Spain; Endocrinology & Nutrition Department, Hospital Universitario La Paz, 28046 Madrid, Spain; Endocrinology & Nutrition Department, Hospital Universitario de Basurto, 48013 Bilbao, Spain; Universidad del País Vasco UPV/EHU, 48013 Bilbao, Spain; Endocrinology & Nutrition Department, Complejo Universitario de Salamanca, 37007 Salamanca, Spain; Endocrinology & Nutrition Department, Hospital Joan XXIII, 43005 Tarragona, Spain; Endocrinology & Nutrition Department, Hospital Reina Sofía, 14004 Córdoba, Spain; Endocrinology & Nutrition Department, Hospital Castellón, 12004 Castellón, Spain; Endocrinology & Nutrition Department, Complejo Universitario de Salamanca, 37007 Salamanca, Spain; Endocrinology & Nutrition Department, Hospital Vall d’Hebron, 08035 Catalunya, Spain; Department of Clinical and Molecular Genetics, Vall d’Hebron Barcelona Hospital Campus, Vall d’Hebron Hospital Universitari, 08035 Barcelona, Spain; Genetic Department, Hospital Universitario Ramón y Cajal, 28034 Madrid, Spain; Department of Medical Sciences, Division of Internal Medicine and Hypertension, University of Torino, 10126 Turin, Italy

**Keywords:** primary aldosteronism, familial hyperaldosteronism, genetic study, pathogenic variant, plasma aldosterone concentration

## Abstract

**Context:**

It is estimated that about 5% of the primary aldosteronism (PA) cases are of hereditary origin (familial hyperaldosteronism, FH). To date, 4 forms of FH have been reported. However, in general little is known about the genetic causes that lead to the development of PA.

**Objective:**

This work aimed to determine the rate of genetic testing for FH in the SPAIN-ALDO Registry and to describe the clinical characteristics of patients with FH. In addition, a literature review of reports of FH cases was performed.

**Methods:**

A retrospective multicenter study of PA in patients followed in 35 Spanish tertiary hospitals (SPAIN-ALDO Registry).

**Results:**

Twenty-five of the 855 patients (3%) with PA included in the registry underwent genetic testing for FH, with complete results available for only 24 patients. However, we found that there were 57 patients who met the criteria for performing a genetic study of PA. Only 8 out of these 57 patients were genetically tested (14.0%), while the reasons to perform a genetic study in the remaining 17 genetically studied cases were quite heterogeneous. A positive result for FH was found in only one case for FH type III (*KCNJ5* pathogenic variant). A systematic review of the literature was performed and identified a total of 25 articles reporting 246 patients with FH type I, 12 articles reporting 72 patients with FH type II, 14 articles reporting 29 cases of FH type III, and 3 articles reporting 12 patients with FH type IV.

**Conclusion:**

The genetic study of FH is often scarce in real-world clinical practice, as 86% of patients with criteria to undergo genetic study were not evaluated in our cohort. Nevertheless, FH is an uncommon cause of PA, representing only 0.2% of cases in the SPAIN-ALDO Registry, although its prevalence may be as high as 4% among suspected cases.

Primary aldosteronism (PA) is the leading cause of secondary hypertension with a prevalence of 10% in the general hypertensive population and up to 20% in refractory hypertensive patients (1, 2). Most cases of PA are sporadic, but about 5% of them are of hereditary origin (familial hyperaldosteronism, FH) (3). To date, 4 forms of FH have been reported. However, little is known about the genetic causes that lead to the development of PA. The first clinical description of FH type I was published in 1966 by Sutherland et al (4) in a father and son whose clinical symptoms of PA were relieved by dexamethasone 2 mg daily. FH-I or glucocorticoid-remediable PA is an autosomal-dominant form of hypertension characterized by increased adrenocorticotropin (ACTH)-dependent aldosterone secretion, renin suppression, and high levels of 18-hydroxycortisol and 18-oxocortisol (2). The genetic alteration involves a genetic crossover between the genes *CYP11B2* encoding aldosterone synthase enzyme, and *CYP11B1*, encoding 11β-hydroxylase enzyme. Thus, aldosterone synthesis is regulated by ACTH rather than angiotensin II (5). FH type II, a non-glucocorticoid–remediable familial form of PA, was first described in 1991 by Gordon et al (6). The genetic defect is localized in the *CLCN2* gene, which encodes for proteins of a chloride channel, ClC2 (7). More recently, FH type III due to a pathogenic variant in *KCNJ5* (8) and FH type IV secondary to pathogenic variants in *CACNA1H* (9) have been described. However, despite these advances in the genetic characterization of PA, few studies have evaluated the true prevalence of FH and analyzed the clinical and hormonal characteristics of these patients (10, 11). In addition, no previous studies have evaluated the rate of genetic testing in a real-life setting, nor the epidemiological and clinical data of FH patients in the Spanish population.

Considering this background, the aim of our study was to determine the rate of genetic test performance for FH in the SPAIN-ALDO Registry and to describe the clinical and hormonal characteristics of patients affected by FH included in our sample. In addition, we have performed a systematic review of FH cases reported in the previous literature.

## Materials and Methods

### Study Population and Definitions

Patients for this study were recruited through the SPAIN-ALDO Registry, which includes patients with PA in follow-up who were enrolled in 34 Spanish tertiary hospitals between January 2018 and July 2023. At the time of data analysis (August 10, 2023), a total of 855 patients with PA had been included in the registry. As we have previously described (12), the clinical data of these patients were entered into an electronic database (REDCap database) (13, 14) after pseudonymization using an identification number (record_Id). Information on demographic characteristics, comorbidities, biochemical and radiological parameters, physical evaluation, and treatments for PA are included in the registry (15).

The study was conducted according to the guidelines of the Declaration of Helsinki and approved by the ethics committee of the Hospital Universitario Ramón y Cajal, Madrid, Spain (approval date: November 10, 2020; code: ACTA 401).

Patient consent was waived due to the retrospective nature of the study. Informed consent was requested only from patients who continued follow-up or who were prospectively included. Written informed consent for the genetic study was obtained from all patients who underwent genetic testing.

### Diagnosis of Primary Aldosteronism and Subtyping

PA was diagnosed according the criteria proposed by the latest clinical European guidelines for PA (1, 2, 16). For subtyping, 336 of 855 patients underwent adrenal venous sampling (AVS), which was successful in 195 individuals. AVS results with (n = 241) or without (n = 95) ACTH stimulation were used to classify PA subtypes. Unilateral disease was assumed if the lateralization index of the aldosterone to cortisol ratio was 4.0 or greater on the dominant side compared to the nondominant side while on ACTH stimulation or at least 2-fold higher under unstimulated conditions and AVS was selective in both sides (1, 6). Patients without available successful AVS were considered to have unilateral disease if a complete biochemical cure was achieved after surgery (n = 150). Definitions of biochemical and clinical cure for PA after adrenalectomy were based on the Primary Aldosteronism Surgical Outcome classification criteria (17).

### Familial Primary Aldosteronism Diagnosis and Genetic Study

The following criteria were used for the diagnosis of FH: FH-I was diagnosed by long–polymerase chain reaction (PCR) amplification of the hybrid gene (*CYP11B1/CYP11B2*); FH-II was diagnosed when a gain-of-function mutation in *CLCN2* was detected, diagnosis of FH-III when a gain-of-function mutation in *KCNJ5* was demonstrated, and FH-IV was diagnosed when a pathogenic variant in *CACNA1H* was detected. Only patients with a positive genetic study were included as FH patient cases.

Genetic study was performed by massive sequence (next-generation sequencing) of the *CLCN2*, *KCNJ5*, and *CACNA1H* genes, using a custom capture kit (Cell3 Target Custom Panel by Nonacus, RRID requested), and sequenced on an Illumina NextSeq 550 System (RRID:SCR_016381). The detection of the hybrid gene *CYP11B1/CYP11B2* was performed by long-PCR amplification using a custom capture kit (TaKaRa LA Taq Hot Start Version).

### Search Strategy for the Identification of Familial Hyperaldosteronism Cases

**Figure 1.**
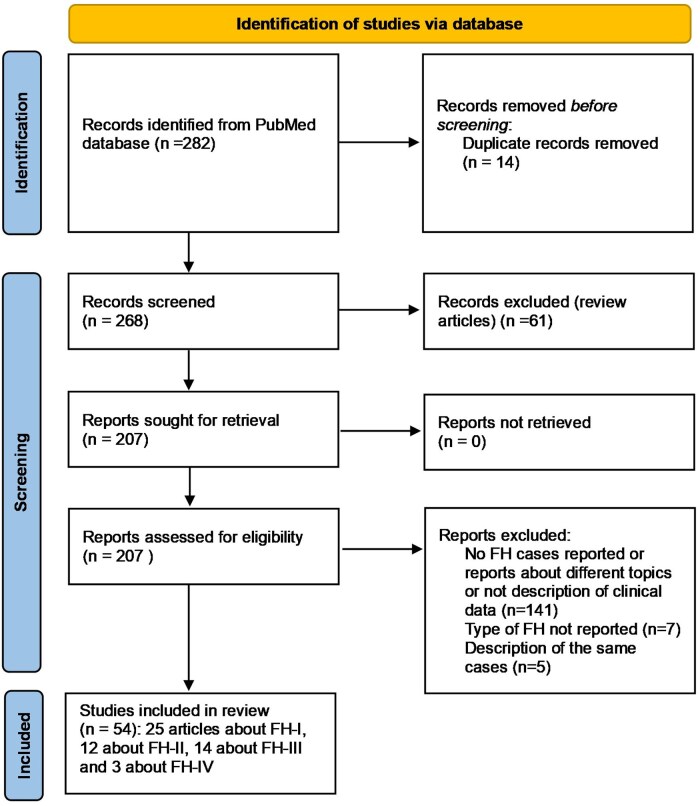
Flowchart for identification of familial hyperaldosteronism (FH) cases.

We followed the SANRA scale (18) for the identification of the previous cases of FH published in the literature. The search strategy was carried out in PubMed without date filter up to August 12, 2023. The following search terms were used: *familial primary aldosteronism* [TI]: 136 results; *familial hyperaldosteronism* [TI]: 61 results; *hereditary aldosteronism* [title]: 18 results; *inherited primary aldosteronism* [TI]: 16 results; *dexamethasone-suppressible aldosteronism*: 2 results; and *glucocorticoid-remediable aldosteronism* [TI]: 49 results. Potentially relevant articles were retrieved after reading the title, abstract, or the whole article and discarding repeated articles. Only articles published in English were included. The articles identified by these searches and relevant references cited in those articles were reviewed. After that, 54 original articles were included: 25 articles about FH-I (10, 19-42), 12 about FH-II (7, 10, 11, 43-51), 14 about FH-III (8, 50, 52-62) and 3 about FH-IV (9, 63, 64) (Fig. 1). The definition of FH-II in the different articles was based on the demonstration of a gain-of-function mutation in *CLCN2* (FH-II genetically proven) or in familial history (when at least 2 members of the same family had confirmed PA; clinical FH-II). For FH-I, III, and IV, we have included only cases with a positive genetic study for *CYP11B1/CYP11B2*, *KCNJ5*, and *CACNA1H*, respectively.

### Statistical Analysis

All statistical analyses were performed with STATA.15. All data are expressed as the mean and SD for normally distributed variables and as median (and range) for nonnormally distributed variables.

## Results

### Baseline Characteristics

A total of 855 patients with PA were included in the study. Of the 383 patients with available AVS results and/or who underwent adrenalectomy, 312 were classified as having unilateral PA and 71 as having bilateral PA. The mean age of the cohort was 56.2 ± 6.04 years and 58.6% (n = 501) were men.

### Rate of Genetic Testing Performance and Description of Familial Hyperaldosteronism Cases

Only 25 of the 855 patients (3.0%) underwent FH genetic testing, of whom results were available in only 24 cases, since the results were still pending in one patient. However, when we evaluated how many patients met the criteria to perform a genetic study of PA, we identified a total of 57 cases (Table 1).

**Table 1.** Patients with indication for genetic study for familial primary aldosteronism and patients who underwent genetic study

Indication of genetic study	Cases not tested	Cases tested for FH*^a^*	Total No. of cases with PA
PA diagnosis before age 30 y	4	0	4
Hypertension diagnosed before age 20 y	20	3	23
PA diagnosis before age 30 y and hypertension diagnosed before age 20 y	4	1	4
First-degree family member with PA	13	5	18
Cerebrovascular hemorrhages before age 40 y	8	0	8
Total of potentially candidates for FH genetic study	49	9	57

Abbreviations: FH, familial hyperaldosteronism; PA, primary aldosteronism.

^*a*^A total of underwent FH genetic testing. Eight out of the 25 cases had indication for genetic study, while in the other 17 cases the genetic study was performed for different clinical reasons (hypertension diagnosis before age 40 years but older than 30 years (n = 8) family history of hypertension in more than 2 relatives [n = 5] and other reasons in the remaining 4 cases).

In the 25 patients who underwent genetic testing, the reasons for performing the study were the following: i) hypertension diagnosis before age 40 years but older than 30 years (n = 8); ii) hypertension diagnosis before age 30 years but older than 20 years (n = 1), iii) hypertension diagnosis before age 20 years (n = 3), family history of hypertension in more than 2 relatives (n = 4), family history of PA (n = 5), and other reasons in the remaining 4 cases.

Of the 24 patients tested with available genetic results, FH was detected in only 1 patient, representing 4.2% of the total evaluated cohort. This patient case corresponded to a Japanese boy diagnosed with PA at age 4 years had no history of hypokalemia but had high plasma aldosterone concentration (PAC) (>90 ng/dL) and left ventricular hypertrophy at diagnosis. He is in treatment with spironolactone (50 mg daily). The genetic study showed a pathogenic variant in *KCNJ5* (variant chr11-128781620 G > A; NM_000890.5:c.452G > A .p.(Gly151Glu)). The functional studies demonstrated that this pathogenic variant alters the functionality of the potassium channel Kir3.4, leading to an abnormal ion current with loss of potassium selectivity, sodium influx, and consequent increased intracellular calcium that causes excessive aldosterone biosynthesis. The mother of this patient is a 46-year-old Japanese woman, with PAC at diagnosis of the PA of 25 ng/dL and normal serum potassium levels. She is on treatment with spironolactone 25 mg/day, with well-controlled hypertension.

### Literature Review of Familial Hyperaldosteronism-I Cases

A total of 25 articles were identified in which 246 patients with FH type I were reported and clinical and/or hormonal data were available. In addition, 1 article describing 165 patients included in the international FH type I database was revised. However, these patients were excluded from our analysis since most of them had been also included in the aforementioned 25 articles. The median age of the FH I cases was 16.5 years (range, 0.5-65 years) and 52.4% were female (n = 129/246). Only 15.1% of the cases had hypokalemia, the median serum potassium levels being 3.86 mEq/mL (range, 2-6.9 mEq/mL) (Table 2). These findings were similar to those described in the GRA international study (n = 165) (65): mean age of 34.1 ± 18.8, 48% women, and mean serum potassium levels of 4.1 ± 0.54 mEq/L.

**Table 2.** Clinical and hormonal features of patients with familial hyperaldosteronism

	FH-I (n = 246)	FH-II (n = 18)	Clinical FH-II*^a^* (n = 60)	FH-III (n = 29)	FH-IV (n = 12)
Age, y	16.5 (0.5-65)	15.5 (0.2-36)	43 (0.6-72)	4 (0.2-62)	20 (0.2-51)
Female, %	52.4% (n = 129)	61.1% (n = 11)	48.2% (n = 26/54)	75.9% (n = 22)	33.3% (n = 4)
SBP, mm Hg	153.5 (90-231)	135 (94-280)	156 (115-200)	150 (120-230)	153.5 (110-192)
DBP, mm Hg	95 (40-124)	95 (62-188)	97 (70-122)	94.5 (70-140)	96 (70-144)
% Hypokalemia	15.1% (n = 18/119)	52.9% (n = 9/17)	54.2% (n = 26/48)	89.3% (n = 25/28)	58.3% (n = 7)
Serum K, mEq/L	3.86 (2-6.9)	3.4 (1.8-4.4)	3.6 (2.3-4.5)	2.65 (1.4-4.4)	3 (1.7-4.1)
PAC, ng/dL	17.02 (2.5-73.2)	26.4 (9.48-100)	20 (12-214.6)	70.7 (23-297)	31.8 (20-119.83)

Abbreviations: DBP, diastolic blood pressure; FH, familial hyperaldosteronism; PAC, plasma aldosterone concentration; SBP, systolic blood pressure.

^*a*^Clinical FH-II refers to those cases with familial history of PA but without demonstration of the *CLCN2* pathogenic variant.

### Literature Review of Familial Hyperaldosteronism-II, -III, and -IV Cases

We identified 12 articles reporting 92 patients with FH type II, of whom 72 patient cases had available clinical and/or hormonal data. Nevertheless, only in 18 out of the 72 cases of FH-II was a pathogenic variant of *CCLN2* detected, while in the other 54 cases the diagnosis was based on familial history of PA. In 10 cases a linkage of the disease locus with the 7p22 was described, and in the remainder the diagnosis was based on family history of PA. A total of 14 articles reporting 29 cases of FH type III and 3 articles describing 12 patients with FH type IV have been found with our search strategy. Clinical and hormonal data are described in Table 2.

## Discussion

Our study reveals that FH is an underinvestigated condition since in our cohort only 25 out of the 855 patients (3.0%) underwent genetic testing and only 8 of the 57 patients (14%) with criteria for genetic study were evaluated. The criteria to undergo genetic testing in the other 17 patients were heterogeneous. Our study is the first that evaluates the rate of genetic testing and the reasons for its evaluation in patients with PA, revealing a view of this topic from real-world practice.

Despite the fact that PA is the most frequent cause of endocrine arterial hypertension, it is still an underdiagnosed condition (66). Our study demonstrates that the investigation of genetic forms of PA is also rarely performed. This situation may be explained by the lack of knowledge about the possibility that PA is of genetic origin and the lack of uniformity regarding the criteria for indicating screening for genetic causes in pulmonary arterial hypertension in general. Although a recent European Reference Network on Rare Endocrine Conditions practical guideline has been published with the aim to guide clinicians when genetic study should be performed in PA, the real situation is that PA remains underdiagnosed in general, and even more so FH (67). PA study should include not only subtyping but also detection of genetic/familial cases to allow the early detection of affected patients and their families (68). Diagnosis of genetic PA patients is important because FH cases are bilateral, and AVS should not be performed. In addition, a specific and effective treatment for FH-I exists based on glucocorticoids to control blood pressure and to reduce the negative effects of the aldosterone excess (1).

A low prevalence of FH was detected in the tested patients since only 1 out of the 24 evaluated patients (4%) met criteria for FH. This prevalence is in accordance with that reported in the PATOGEN study (7%) if we considered only the patient cases who underwent genetic testing (10). That study is the largest evaluating PA genetically: A total of 300 consecutive PA patients were tested by long-PCR of the *CYP11B1/CYP11B2* hybrid gene, and only 2 families had FH-I (0.66%); however, another 12 families met the clinical criteria for FH-II (6%). When the researchers studied the relatives of these patients, a total of 21 additional cases of FH-I and 15 of FH-II were detected. Nevertheless, we should take into account that the diagnosis of FH-II was based only on family history of PA because this study was published before the discovery of the underlying genetic alteration of FH-II. Thus, it is possible that some of the cases classified as FH-II were actually sporadic cases of PA in the same family. In agreement with this hypothesis, the prevalence of FH in a German registry (11) was 1.2% (2/166 patients), all with clinical FH-II (but the diagnosis was based only on familial history of PA, without demonstration of pathogenic variants in the *CLCN2* gene), and in an Australian study 0.36% for FH-I and 2.8% for FH-II (also based only on familial history of PA, without demonstration of pathogenic variants in the *CLCN2* gene) (44). Differences across these studies may be justified because in our study, only patients with a high suspicion of FH were included, whereas in the German study (11), patients with apparently sporadic PA were tested. Nevertheless, in the Italian study (19), consecutive patients with a diagnosis of PA were included, and those referred to their unit with suspicion of FH were excluded from the study.

Based on the literature review, we found that FH-I is the more frequent type of FH, with a total of 246 cases reported in the literature. As we describe in the Table 2, although FH-II patients are older than type I patient cases (median age, 43 vs 16.5 years), these differences disappear when only genetically confirmed case are considered. Nevertheless, independently of the criteria used for the definition of FH-II, the prevalence of hypokalemia was significantly higher than in FH-I (50% in contrast to in 15% of the FH-I cases). Some previous authors have suggested that the phenotype of type II cases is similar to sporadic cases, even though familial cases of unilateral PA have been described in these families (11, 44, 48, 49, 51). Furthermore, even in the same family members, some patients had unilateral PA and others bilateral PA (49). Nevertheless, this last observation is probably related to the fact that before the discovery of the *CLCN2* mutation as the cause of FH-II, all cases with family history of PA without FH-I were classified as FH-II. Thus, it is very likely that some sporadic cases occurring in the same family were classified as type II when they were not really genetic cases. Although the diagnosis of FH-I and FH-II is genetic, there are some clinical and hormonal data that may orientate to the diagnosis. For example, patients with FH-I are characterized by a positive response of aldosterone to dexamethasone administration and high urinary levels of 18-oxocortisol and 18-hydroxycortisol; and FH-II usually exhibits very high urinary 18-oxocortisol and 18-hydroxycortisol levels and severe hypokalemia. However, this indirect testing may be misleading in patients with FH (69).

The descriptions of FH type III and IV cases are limited to the report of small series of cases, with only 29 and 12 cases described in the literature, respectively. FH-III was first documented in 2008 (8), and is frequently associated with severe hypertension, hypokalemia, and very high concentrations of aldosterone. Bilateral adrenalectomy is generally necessary to achieve proper blood pressure control in these patients (17/29 described cases required bilateral adrenalectomy) (8, 54, 55, 57-59, 61, 62, 70). One of the main clues to suspect FH-III is the severity of the PA and the pediatric age of diagnosis (median age of 4 years). In fact, all the reported cases except for one patient case diagnosed at age 62 years were younger than 20 years. The 62-year-old woman had a p.Y152C germline mutation in *KCNJ5,* associated with a mild phenotype (58). FH-IV is diagnosed by demonstrating a pathogenic variant in the *CACNA1H* gene. Its clinical presentation is variable since some patient cases are diagnosed with severe hypertension at age 2 months (9), and others present with milder PA forms detected at age 50 years (64). The prevalence of hypokalemia was similar to that described in our sporadic cases (58.3% vs 59.2%). Thus, the diagnosis of FH-IV is mainly based on genetic study and should be suspected in young patients with a familial history of PA, after excluding FH-I.

In our recent study comparing familial and sporadic PA cases (71), we found that in general patients with FH are younger and have a family history of PA more frequently than sporadic PA cases. In addition, FH-I cases are characterized by a low prevalence of hypokalemia and FH-III by a severe aldosterone excess causing hypokalemia in more than 85% of patients. However, the clinical and hormonal profiles of FH-II and sporadic cases were similar, except for a younger age and higher diastolic blood pressure in the FH-II group. Similarly, the clinical and hormonal phenotypes of FH type IV and sporadic cases were comparable, apart from a lower age and serum potassium levels and higher plasma renin activity at presentation in the former. However, these clinical and hormonal data are not specific to FH, which leads to the need to perform a genetic study in all patients with clinical-hormonal suspicion of FH to establish a certain diagnosis.

While we consider that the findings of our study are important, we are aware that it has several limitations. First, its retrospective design is associated with inherent limitations. Another weakness of the study is the fact that few patient cases underwent genetic study. In addition, of the patients genetically evaluated, results were not available in 36% (9/25) of the patient cases. Regarding the search for FH-II cases, we are aware that the reported cases of FH-II include also non-*CLCN2*–mutated patients, underlining the fact that many of these families could in reality be sporadic PA in the same family. However, despite these limitations, we consider that our results are important since they represent what occur in the real-life practical setting in Spain.

### Conclusion

Genetic studies of FH are often scarce in real-world clinical practice, since 86% of patients with criteria to perform a genetic study were not evaluated in our cohort. Yet FH is an uncommon cause of PA, representing only 0.1% of cases in the SPAIN-ALDO Registry, although its prevalence can increase up to 8% among suspected cases studied.

## Data Availability

Some or all data sets generated during and/or analyzed during the current study are not publicly available but are available from the corresponding authors on reasonable request.

## Abbreviations

ACTH: adrenocorticotropin
AVS: adrenal venous sampling
DBP: diastolic blood pressure
FH: familial hyperaldosteronism
PA: primary aldosteronism
PAC: plasma aldosterone concentration
PCR: polymerase chain reaction
SBP: systolic blood pressure
